# Dr. Louis A. Duhring

**Published:** 1913-06

**Authors:** 


Dr. Louis A. Duhring died in Philadelphia on May 8 at the age of 67 years. He was graduated from the medical department of the University of Pennsylvania in the class of 1867, subsequently serving for fifteen months as interne in the Philadelphia Hospital. Later he went abroad, spending two years in study in Paris, London and Vienna. On returning to Philadelphia in 1870 he established a dispensary for the study and treatment of diseases of the skin, serving as attending physician for a period of seven years, and later becoming consulting physician. In 1871 he was made clinical lecturer on diseases of the skin in the University of Pennsylvania, and in 1876 he was advanced to the professorship of that branch, serving until 1911, when he was made emeritus professor. He was a Fellow of the College of Physicians of Philadelphia, a member and one of the founders of the American Dermatological Association, honorary member of the London, Vienna, and Italian Dermatological Societies, corresponding member of the New York, French and German Dermatological Societies. He was the author of several formal publications dealing with diseases of the skin and a liberal contributor to the literature of the subject.—*Medical Record.*

